# The comparison of meat yield, quality, and flavor between small-tailed Han sheep and two crossbred sheep and the verification of related candidate genes

**DOI:** 10.3389/fnut.2024.1399390

**Published:** 2024-08-01

**Authors:** Cheng Xiao, Yu Liu, Wenjun Zhao, Yingjia Liang, Chao Cui, Shaoying Yang, WenWen Fang, Lisheng Miao, Zhiyu Yuan, Zihan Lin, Bo Zhai, Zhongli Zhao, Lichun Zhang, Huihai Ma, Haiguo Jin, Yang Cao

**Affiliations:** ^1^Institute of Animal and Veterinary Sciences, Jilin Academy of Agricultural Sciences, Gongzhuling, China; ^2^Research Institute for Farm Animal Biology (FBN), Institute of Muscle Biology and Growth, Dummerstorf, Germany; ^3^Institute of Agricultural and Environmental Sciences, Rostock University, Rostock, Germany; ^4^College of Agriculture, YanBian University, Yanji, China; ^5^College of Plant Protection, Jilin Agricultural University, Changchun, China

**Keywords:** small-tailed Han sheep, crossbred sheep, meat yield, quality, volatile compound, *PDK4* gene, *TMEM273* gene

## Abstract

**Introduction:**

In Northeast China, Dorper and Australian White rams are commonly crossbred with small-tailed Han (STH) ewes to improve the offspring's meat yield and quality. However, the differences in traits and the flavor between the crossbred sheep and STH sheep remain unclear. In addition, the candidate genes potentially influencing the meat quality in the three sheep breeds require further verification.

**Methods:**

A total of 18 2-month-old healthy rams were raised over a period of 5 months, which included 6 STH, 6 Dorper and small-tailed Han crossbred (Do × STH), and 6 Australian white and small-tailed Han crossbred (Au × STH) offspring. The differences in slaughter, meat quality traits, fatty acid and amino acid composition in the muscular *longissimus dorsi* (MLD), and volatile compounds in the semitendinosus muscle were compared between the sheep breeds. The candidate genes related to intramuscular fat (IMF) content and fatty acids were validated.

**Results:**

The results of this study revealed that the crossbred sheep had higher body weight, carcass weight, bone weight, net meat weight, and IMF content than the STH sheep (*p* < 0.05). The Do × STH offspring had a higher pH value (24 h), moisture content, and cooking percentage; they also had redder and brighter meat color. The content of myristate, palmitic, and margaric acids in the crossbred sheep was higher than that in the STH sheep (*p* < 0.05). The Do × STH offspring had the highest saturated fatty acid content (*p* < 0.05). The Au × STH offspring had the highest protein content (*p* < 0.05). The arachidonic acid and amino acid (Asp, Ala, Ile, Leu, Lys, Thr, and essential amino acid) contents were higher in the STH sheep than in the crossbred sheep (*p* < 0.05). The odor activity value (OAV) analysis showed that most of the aldehydes in the Au × STH offspring had higher values. The *PDK4* gene expression was positively associated with the IMF content and was negatively correlated with the linoleic acid content in the Do × STH sheep (*p* < 0.05). The *TMEM273* gene expression was positively associated with linoleic and arachidonic acid contents and was negatively correlated with oleic and palmitic acid contents in the Do × STH sheep (*p* < 0.05).

**Discussion:**

The results showed the differences between the crossbred sheep and STH sheep and provided the candidate genes related to meat quality in sheep.

## Introduction

With the rise in economic levels and the changes in dietary habits of society, mutton has become popular with consumers for its tenderness, flavor, and nutrition. Small-tailed Han (STH) sheep are widely raised in northeast China due to their high fecundity and environmental acceptability (1). However, the meat yield and quality of STH sheep are lower than that of Dorper and Australian White sheep, and they cannot meet the demands of the local markets (2). Dorper sheep, a South African breed, have high meat yield and quality (3). Australian White sheep are also a famous breed with high meat quality (4). In Northeast China, these two breeds, Dorper and Australian White sheep, are commonly crossbred with STH sheep to improve the meat yield and quality of the offspring. The flavor of mutton is considered a vital factor affecting consumers' purchase decisions. The composition of fatty acids, amino acids, and inosinic acids in mutton plays a significant role in providing rich taste and synthesizing a variety of volatile compounds that affect the odor of mutton. These compounds are produced through the Maillard and Strecker reaction (5). The differences in slaughter and meat quality traits, fatty acid and amino acid composition, and volatile compounds between the crossbred sheep and STH sheep remain unclear, and addressing how to continuously improve the meat quality in crossbred sheep has become a crucial problem.

Many factors affect the quality traits of mutton, including breed, gender, genetics, nutrition, and environment (6–10). Among these traits, genetics plays a vital role in determining mutton quality traits under the same feeding condition. Lipid metabolism-related regulatory genes or proteins affect adipocyte differentiation and fat accumulation and regulate intramuscular fat (IMF) content and meat quality in livestock, such as *PPARG, CEBPA*, and *LPL*, among others (11–13). However, these genes in various breeds or species may not have similar functions of phenotype regulation. For example, the *PPARG* gene expression or its existing single nucleotide polymorphism (SNP) sites are associated with the IMF content in cattle (14), but there is no relationship with other breeds or species (15, 16). Thus, exploring novel candidate genes related to the IMF content or fatty acid composition in certain breeds is an efficient way to improve meat quality at the molecular plane.

*Pyruvate dehydrogenase kinase 4* (*PDK4*) inhibits pyruvate dehydrogenase complex (PDC) activation to determine which glucose or fatty acid oxidation provides body energy (17) and affects lipid metabolism (18). A study reported that an SNP site at the ninth intron of the *PDK4* gene was associated with the IMF content in pigs (19). Another study found that the *PDK4* gene expression increased in pigs with high IMF content and chickens with high body weight (20). The association of the *PDK4* gene with the IMF content or fatty acid composition in sheep remains unknown. Trans-membrane family proteins promote the interaction of the endoplasmic reticulum and the mitochondria membrane and affect lipid droplet formation and accumulation (21), of which the *TMEM41B* gene participates in lipid droplet formation (22). The *TMEM273* gene, a member of the trans-membrane family, has a molecular structure similar to that of other proteins in the family. However, fewer studies concerning the *TMEM273* gene and lipid metabolism have been reported. Therefore, this study aimed to compare the differences in slaughter and meat quality traits, fatty acid and amino acid composition, and volatile compounds between the crossbred sheep and STH sheep. Additionally, it sought to explore whether the *PDK4* or *TMEM273* gene expression in the *longissimus dorsi* muscle correlates with the IMF content or fatty acid composition in sheep. The results of this study may promote an understanding of the differences between crossbred sheep and STH sheep and provide new insights into improving the IMF content or meat quality in sheep.

## Materials and methods

### Animals

Six 2-month-old healthy male small-tailed Han (STH) sheep, six F1 generation of Dorper and small-tailed Han crossbred sheep (Do × STH sheep), and six F1 generation of Australian white and small-tailed Han crossbred sheep (Au × STH sheep) were obtained from the Jilin Academy of Agricultural Sciences. They were raised over a period of 5 months in independent pens under a constant temperature condition and fed the same diet recommended by the “China meat sheep feed standard.” The sheep were injected with the required medicine to expel parasites, and their feeding conditions were adjusted for a week before the experiments began. The sheep were fed twice a day with concentrates and hay at a ratio of 4:6 and were given free access to water. After 5 months, the sheep were made to fast for 12 h, after which they were stunned with a pistol, hung, bled, eviscerated, and split. The muscular *longissimus dorsi* (MLD) was sampled to measure the meat quality traits, fatty acid composition, and amino acid composition; the semitendinosus muscle was sampled to detect the composition of volatile compounds. All procedures involving animals, as well as welfare and ethical considerations, were approved by the Committee for the Ethics of Animal Experiments under AWEC 2019A05 on 16 May 2019.

### Slaughter traits

The body weight of the sheep was weighed and recorded after a 12-h fast. After slaughter, the weights of the head, hooves, carcass, *longissimus dorsi* muscle, tare, liver, heart, lungs, spleen, kidneys, tail fat, and gut fat were measured and recorded. Then, the separated meat and bone were used to measure the net meat weight and calculate the net meat percentage, dressing percentage, and bone/meat ratio.

### Intramuscular fat content and meat quality trait measurement

After slaughter, the muscular *longissimus dorsi* (MLD) was taken from the left side at the 12th and 13th rib area 45 min postmortem to measure the intramuscular fat (IMF) content and other meat traits. The IMF content was measured using a Soxhlet extractor method in accordance with the Chinese standards for the measurement of fat content within food (GB 5009.3-2016). The sample included 5 g of ground meat, and petroleum ether was used as an organic solvent (Sigma-Aldrich, 32248, St. Louis, MO, USA). The meat color was described using three different color parameters, including brightness (L^*^), redness (a^*^), and yellowness (b^*^), at 1 h and 24 h postmortem, following the method described by Costa et al. (23). A sample of 100 g of meat was hung for 24 h and 48 h under a constant temperature condition to record the remaining weight and calculate the drip loss percentage. A sample of 100 g of meat without adipose and connective tissues was cooked to calculate the cooking percentage following the method described by Mao et al. (24). The share force, moisture content, and pH value (24 h) of the sample were measured following the method described by Mao et al. (24). The measurement of protein content and the analysis of texture were performed by the Jilin Ministry of the Agriculture and Rural Quality Testing Center (Changchun, China). All trial samples had three biological replicates.

### Fatty acid composition detection

A 2-g of the sample from the MLD was homogenized with 2 mL of nonadecanoic acid, an internal standard, and then 10 mL of hydrochloric acid was added to the sample; the mixture was incubated in a water bath pot in a flask at 70°C for 40 min. The hydrolysate was placed in another flask, and 25 mL of methyl ether and petroleum ether were added to the mixture at a ratio of 1:1 (Sigma-Aldrich, 32248, St. Louis, MO, USA). Then, the mixture was concentrated into a powder using a rotary evaporator (Great Wall, R-2050Ex, Zhengzhou, China) at 40°C. The powder was treated with 8 mL of 2% sodium methoxide and 7 mL of 15% boron trifluoride to form fatty acid methyl esters (FAME), and it was extracted with 20 mL of n-heptane to analyze fatty acid composition using a capillary gas chromatograph and mass spectrometer (Agilent 7890A-5975C GC/MS, Santa Clara, CA, USA) with a column (HP-5MS, 60 m × 250 μm × 0.25 μm, Santa Clara, CA, USA). The detailed procedure was conducted according to the Chinese standards for the measurement of fatty acid composition within foods (GB 5009.168-2016) and following the method described by Kalbe et al. (25).

### Amino acid composition detection

A 300 mg of meat sample from the MLD was homogenized and dissolved in 10 mL of 6 mol/L HCl Sigma-Aldrich, 32248, St Louis, MO, USA) and 0.1% phenol (Sigma) at 110°C for 22 h. The hydrolysate was concentrated into a powder using a rotary evaporator (Great Wall, R-2050Ex, Zhengzhou, China) at 40°C. Then, 2 mL of sodium citrate (Sigma), with a pH value of 2.2, was used to dilute the powder for analyzing amino acid composition using the liquid chromatograph and mass spectrometer (Agilent, 6500 LC/MS). The detailed procedure was conducted according to the Chinese standards for the measurement of amino acid composition within foods (GB 5009.124-2016) and following the method described by Belhaj et al. (26). Norleucine is the internal standard. All trial samples had three biological replicates.

### Detection of volatile compounds

A 1.5 g of ground meat sample from the semitendinosus muscle in a 20 mL sealed extraction flask (Supelco, 57330-U) with 5 mL of NaCl and 50 μL of 0.05 mg/mL trimethylpyridine (Sigma), an internal standard, was subjected to magnetic stirring at 500 rpm speed in an 85°C water bath pot for 40 min. The extraction catheter (Supelco, 57348-U) collected volatile flavor substances from the sample in the extraction flask into the gas chromatograph and mass spectrometer (Agilent 7890A-5975C GC/MS, Santa Clara, CA, USA) with a HP-5MS column (60 m × 250 μm × 0.25 μm, Santa Clara, CA, USA) to analyze the volatile compounds. Helium, a carrier gas, flows at a speed rate of 1.0 mL/min, at an input device temperature of 250°C, at a pressure of 14.87 Pa, and at a split ratio of 1:1. The heating conditions were as follows: 40°C for 3 min, heated to 150°C at a rate of 4°C/min and held for 1 min, further heated to 200°C at a rate of 5°C/min, and then heated to 230°C at a rate of 20°C/min and held for 5 min. The ion source temperature was set at 250°C, the MS transmission line temperature was set at 250°C, and the MASS scanning range was m/z 30 ~ 400. An odor activity value (OAV) was calculated according to the formula, OAV = Compounds concentration/its odor threshold, to reveal the vital volatile compounds in the different sheep breeds. Higher OAV values of compounds play a crucial role in affecting the meat odor.

### Total RNA isolation, cDNA reverse, and qPCR verification

The total RNA of the sample from the MLD was isolated using the TRIzol reagent following the manufacturer's instruction (Thermo Fisher Scientific), and then, the RNA was reversed into cDNA following the procedure described in a previous study (27). The expression of the *PPARG, PDK4*, and *TMEM273* genes was detected by conducting a qPCR test using the Roche LightCycler^®^ 480 (Roche Applied Science) device, following the protocol described in a previous study (27), and considering the *GAPDH* gene as the reference control. The primer sequences were synthesized by GenePharma Company, Shanghai (Table 1). All trial samples had three biological replicates.

**Table 1:** Primer sequences for the qPCR test.

**Genes**	**Primer sequence 5^′^ → 3^′^**	**Product length/bp**	**Gene ID**
PDK4	F: 5′-GAAAACGCATGTGAAAGAACCTC−3′	198 bp	XM_004007738.6
	R: 5′-CTTTTGGTCCTCTGGGCTTTT−3′		
PPARG	F: 5′-CCGTGGACCTTTCTATGATGG-3′	193 bp	NM_001100921.1
	R: 5′-TACAGGCTCCACTTTGATTGC-3′		
TMEM273	F: 5′-AGAACGCTGACTGTCCTCCTC-3′	128 bp	XM_060406374.1
	R: 5′-ATGGCGATGCCCACAGCA-3′		
GAPDH	F: 5′-TCCACGGCACAGTCAAGG-3′	228 bp	NM_001190390.1
	R: 5′-CACGCCCATCACAAACAT-3′		

### Statistical analysis

The experimental data were described as mean ± SEM and were analyzed using SPSS 17.0 software. The Student's *t*-test model was used to compare the data of two groups, while the one-way ANOVA model was used to compare the data of multiple groups. The correlation of the IMF content and fatty acid composition in all samples was compared first. Then, the gene expression, the IMF content, and the fatty acid composition in each group were compared using the Pearson model (paired) in SPSS 17.0 software.

The figures were illustrated using GraphPad Prism 6.0 software. Statistical significance levels were set at a *p*-value of < 0.05. Original images and data are shown in the Supplementary material.

## Results

### The crossbred sheep had higher meat yield than the STH sheep

**Figure 1:**
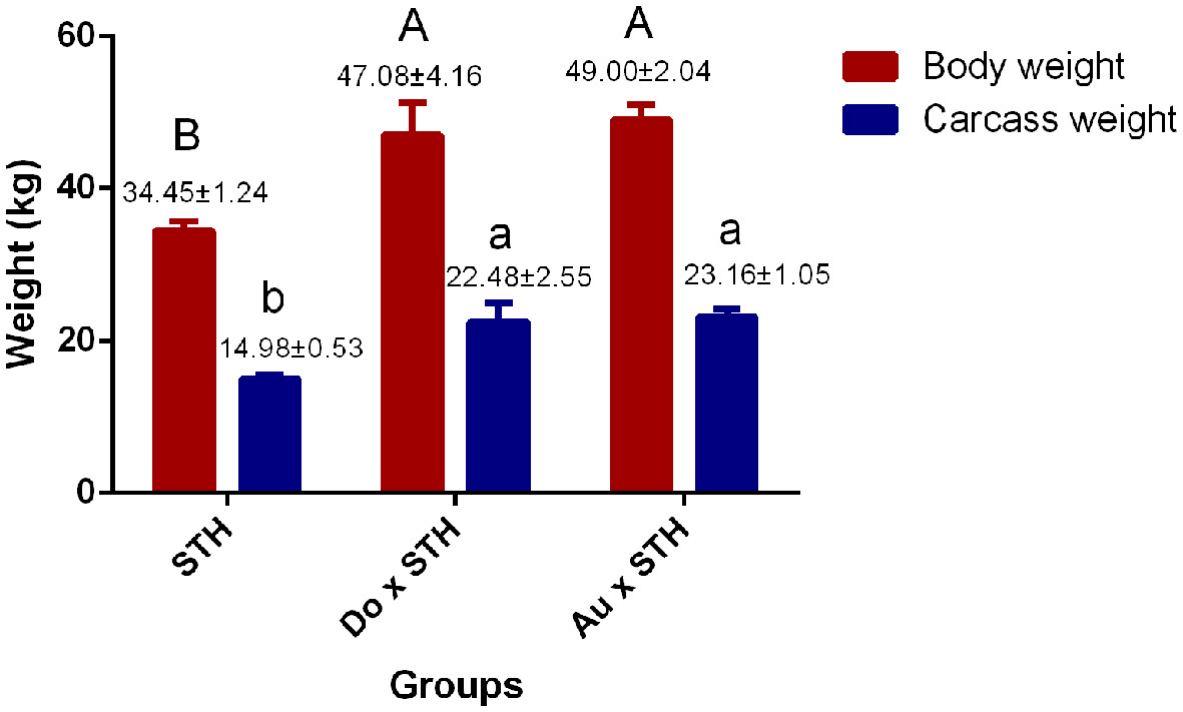
The comparison of the body and carcass weight between the groups. The body and carcass weights in the small-tailed Han (STH) sheep, in the offspring of the Dorper and STH (Do × STH) sheep, and in the offspring of the Australian white and STH (Au × STH) sheep. The different letters represent significant changes (*p* < 0.05).

A total of 18 experimental sheep, raised 5 months later, showed different body and carcass weights. The Do × STH offspring and the Au × STH offspring had higher body and carcass weights than the STH sheep (*p* < 0.05). However, no significant differences were observed between the Do × STH offspring and the Au × STH offspring (*p* > 0.05) (Figure 1). The crossbred sheep had higher net meat weight than the STH sheep (*p* < 0.05) (Table 2). In addition, the weight of the hooves, bones, lungs, spleen, kidneys, and gut fat was higher in the crossbred sheep than in the STH sheep, especially, in the Do × STH offspring (*p* < 0.05). Contrarily, the tail fat weight and the bone/meat ratio in the STH sheep were higher than those in the crossbred sheep (*p* < 0.05). The weight of the head, tare, liver, heart, and the *longissimus dorsi* muscle, the net meat weight, and the dressing percentage had no significant changes between the groups (*p* > 0.05). The results suggest that the crossbred sheep possessed higher meat yield and a faster growth rate compared to the STH sheep during the same feeding period and under the same feeding condition. The details are shown in Supplementary Table 1.

**Table 2:** The slaughter traits between the groups.

**Traits**	**Breeds (Mean** ±**SEM)**		
	**STH sheep (*****n** =* **6)**	**Do** × **STH sheep (*****n** =* **6)**	**Au** × **STH sheep (*****n** =* **6)**
Head weight (Kg)	2.57 ± 0.15	2.28 ± 0.31	2.53 ± 0.05
Hooves weight (Kg)	0.80 ± 0.04^b^	0.98 ± 0.07^ab^	1.05 ± 0.04^a^
Tare weight (Kg)	3.22 ± 0.19	3.78 ± 0.26	3.75 ± 0.28
Bone weight (Kg)	3.33 ± 0.10^b^	4.17 ± 0.37^a^	4.48 ± 0.21^a^
Liver and heart weight (Kg)	0.63 ± 0.02	0.82 ± 0.08	0.78 ± 0.03
Kidney weight (Kg)	0.18 ± 0.03^b^	0.32 ± 0.04^ab^	0.35 ± 0.04^a^
Lung weight (Kg)	0.55 ± 0.04^b^	0.78 ± 0.09^a^	0.7 ± 0.03^ab^
Spleen weight (Kg)	0.04 ± 0.002^b^	0.10 ± 0.01^a^	0.07 ± 0.01^ab^
Gut fat (Kg)	0.09 ± 0.02^b^	0.32 ± 0.07^a^	0.25 ± 0.06^ab^
Tail fat (Kg)	0.40 ± 0.06^a^	0.19 ± 0.03^b^	0.30 ± 0.05^ab^
*Longissimus dorsi* weight (Kg)	1.06 ± 0.06	0.94 ± 0.10	0.99 ± 0.05
Net meat weight (Kg)	11.01 ± 0.45^b^	16.71 ± 1.83^a^	16.78 ± 0.75^a^
Net meat percentage	0.32 ± 0.01	0.35 ± 0.01	0.34 ± 0.01
Bone/meat	0.31 ± 0.01^a^	0.25 ± 0.01^b^	0.27 ± 0.01^b^
Dressing percentage	0.43 ± 0.01	0.47 ± 0.01	0.47 ± 0.01

STH, Small-tailed Han sheep; Do × STH, Dorper and small-tailed Han crossbred; Au × STH, Australian white and small-tailed Han crossbred. ^a, b^different superscript letters indicate significant differences among groups (*P* < 0.05).

### The do × STH offspring had higher IMF content and meat quality traits

**Figure 2:**
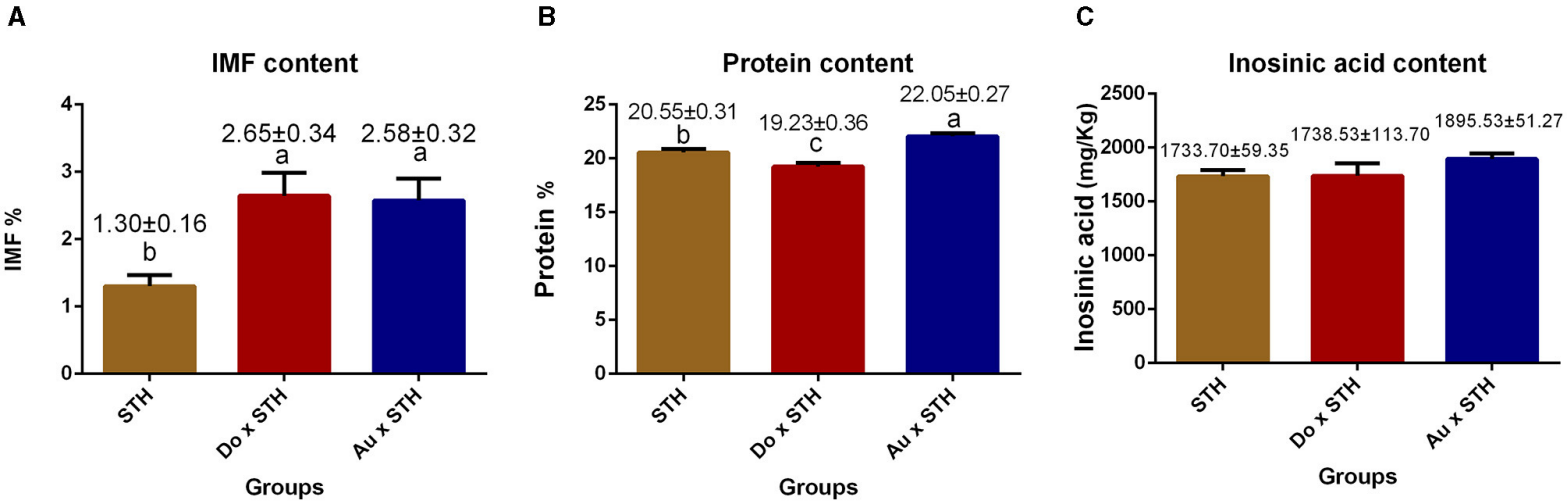
The comparison of the IMF, protein, and inosinic acid content in the MLD between the groups. **(A)** The intramuscular fat content in the STH sheep, in the offspring of the Do × STH sheep, and in the offspring of the Au × STH sheep. **(B)** The protein content in the STH sheep, in the offspring of the Do × STH sheep, and in the offspring of the Au × STH sheep. **(C)** The inosinic acid content in the STH sheep, in the offspring of the Do × STH sheep, and in the offspring of the Au × STH sheep. The different letters represent significant changes (*p* < 0.05).

The crossbred sheep had higher IMF content than the STH sheep (*p* < 0.05), but no significant differences were observed between the Do × STH offspring and Au × STH offspring (*p* > 0.05) (Figure 2A). The Au × STH offspring had the highest protein content, followed by the STH sheep and the Do × STH offspring (*p* < 0.05) (Figure 2B). Inosinic acid influences the freshness and taste of meat (28, 29), but no significant changes were observed in the content between the groups (*p* > 0.05) in this study (Figure 2C). No significant differences were observed in the meat color at 1 h among the groups (*p* > 0.05). At 24 h, the crossbred sheep had a redder meat color than the STH sheep, and the STH sheep had a brighter meat color than the Au × STH sheep (*p* < 0.05) (Table 3). No significant changes were observed in drip loss (24 or 48 h) and shear force between the groups (*p* > 0.05). The Do × STH sheep and STH sheep had higher moisture content and pH values (24 h) than the Au × STH sheep (*p* < 0.05). The Do × STH sheep had a higher cooking percentage than the STH sheep (*p* < 0.05). The meat elasticity was higher in the STH sheep than that in the Do × STH sheep (*p* < 0.05). These results suggested that the Do × STH crossbred sheep group may have higher meat quality traits than the other groups due to their brighter and redder meat color, higher pH values at 24 h, higher IMF content, higher moisture content, and higher cooking percentage. The Au × STH crossbred sheep had higher IMF and protein content, along with a redder meat color; however, the remaining meat traits of the Au × STH crossbred sheep were lower than those observed in the other groups. The details are shown in Supplementary Table 2.

**Table 3:** The meat quality traits between the groups.

**Traits**	**Breeds (Mean** ±**SEM)**		
	**STH sheep (*****n** =* **6)**	**Do** × **STH sheep (*****n** =* **6)**	**Au** × **STH sheep (*****n** =* **6)**
Color (L^*^ 1 h)	36.94 ± 0.60	36.77 ± 0.70	34.60 ± 1.51
Color (L ^*^24 h)	45.02 ± 1.02^a^	43.39 ± 1.09^ab^	41.41 ± 0.23^b^
Color (a^*^ 1 h)	14.29 ± 0.83	15.83 ± 0.82	16.47 ± 1.05
Color (a^*^ 24 h)	14.70 ± 0.72^b^	18.21 ± 0.66^a^	17.09 ± 0.91^a^
Color (b^*^ 1 h)	5.35 ± 0.29	5.51 ± 0.47	5.42 ± 0.47
Color (b^*^ 24 h)	11.25 ± 0.53	12.67 ± 0.31	11.92 ± 0.43
pH value (24 h)	5.80 ± 0.05^a^	5.60 ± 0.07^a^	5.34 ± 0.10^b^
Drip loss 24 h	2.25 ± 0.59	1.67 ± 0.27	1.31 ± 0.24
Drip loss 48 h	2.95 ± 0.55	2.70 ± 0.35	1.98 ± 0.34
Shear force (N)	33.83 ± 9.82	39.03 ± 6.06	49.01 ± 7.18
Moisture content (%)	77.14 ± 0.31^a^	76.14 ± 0.38^a^	73.22 ± 0.59^b^
Cooking percentage (%)	54.71 ± 0.70^b^	57.35 ± 0.74^a^	55.34 ± 0.90^ab^
Adhesive force (N)	0.10 ± 0.02	0.22 ± 0.06	0.15 ± 0.02
Coherency	0.47 ± 0.01	0.42 ± 0.03	0.43 ± 0.02
Elasticity	0.66 ± 0.01^a^	0.61 ± 0.02^b^	0.64 ± 0.01^ab^
Caking property (N)	−0.13 ± 0.02	−0.25 ± 0.07	−0.17 ± 0.01
Viscidity (N)	26.34 ± 2.39	56.31 ± 14.13	38.34 ± 6.71
Mastication (N)	17.65 ± 1.80	34.35 ± 8.44	24.68 ± 4.45
Hardness (N)	12.04 ± 1.11	22.47 ± 4.98	15.53 ± 2.52

STH, Small-tailed Han sheep; Do × STH, Dorper and small-tailed Han crossbred; Au × STH, Australian white and small-tailed Han crossbred. ^a, b^different superscript letters indicate significant differences among groups (*P* < 0.05).

### Fatty acid profile analysis

Fatty acids are the main components of triglycerides (TG) and play crucial biological functions in adipogenesis and TG accumulation (30). The fatty acid content and composition in mutton affect the flavor, nutrients, and meat quality (31, 32). Therefore, the fatty acid profiles in the muscular *longissimus dorsi* (MLD) were detected, and the results demonstrated that the crossbred sheep had a higher content of myristate, palmitic, and margaric acids compared to the STH sheep (*p* < 0.05) (Table 4). No significant differences were observed between the Do × STH offspring and the Au × STH offspring (*p* > 0.05). The Do × STH offspring had a higher content of saturated fatty acids than the STH sheep (*p* < 0.05). However, the STH sheep had a higher content of arachidonic acid than the Do × STH sheep (*p* < 0.05). No significant differences were observed in palmitoleic acid, stearic acid, oleic acid, or linoleic acid between the groups (*p* > 0.05). Saturated fatty acids and monounsaturated fatty acids are closely correlated with meat flavor (33); the crossbred sheep may have better meat flavor than the STH sheep. The details are shown in Supplementary Table 3.

**Table 4:** The fatty acid composition between the groups.

**Fatty acid categories**	**Fatty acid percentage (mg/10g)**	**Breeds (Mean** ±**SEM)**		
		**STH sheep (*****n** =* **6)**	**Do** × **STH sheep (*****n** =* **6)**	**Au** × **STH sheep (*****n** =* **6)**
Saturated	Myristate	1.26 ± 0.10^b^	1.72 ± 0.04^a^	1.92 ± 0.04^a^
	Palmitic acid	20.45 ± 0.89^b^	23.82 ± 0.70^a^	24.60 ± 0.60^a^
	Margaric acid	0.92 ± 0.06^b^	1.48 ± 0.13^a^	1.33 ± 0.09^a^
	Stearic acid	21.33 ± 0.83	20.43 ± 1.06	19.07 ± 0.85
Monounsaturated	Palmitoleic acid	1.42 ± 0.06	1.51 ± 0.10	1.73 ± 0.06
	Oleic acid	37.10 ± 1.65	38.77 ± 1.27	39.90 ± 0.73
Polyunsaturated	Linoleic acid	12.87 ± 2.05	8.78 ± 1.10	8.34 ± 0.59
	Arachidonic acid	3.80 ± 0.52^a^	2.14 ± 0.37^b^	2.38 ± 0.28^ab^
Saturated fatty acid		43.96 ± 1.01^b^	47.45 ± 1.15^a^	46.92 ± 0.50^ab^
Monounsaturated fatty acid		38.52 ± 1.70	40.27 ± 1.31	41.03 ± 0.78
Polyunsaturated fatty acid		16.67 ± 2.57	10.91 ± 1.47	10.72 ± 0.85

STH, Small-tailed Han sheep; Do × STH, Dorper and small-tailed Han crossbred; Au × STH, Australian white and small-tailed Han crossbred. ^a, b^different superscript letters indicate significant differences among groups (*P* < 0.05).

### Amino acid profile analysis

The composition of amino acids affects the fatty acid content, meat color, and pH value (34). Additionally, these acids synthesize aromatic aldehydes through the Strecker reaction, which affects the flavor of meat (35). They also possess different tastes; for example, Glu, Arg, Asp, and Gly possess an umami taste, while Gly Ser, Thr, Lys, Pro, and Ala have a sweet taste (36). The amino acid profile was detected to identify the taste between the groups. Interestingly, the STH sheep showed a higher content of amino acids than the crossbred sheep, including Asp, Thr, Ala, Ile, Leu, and Lys (*p* < 0.05) (Table 5), and these amino acids may affect the taste of meat. In addition, the STH sheep had a higher content of essential amino acids than the Do × STH sheep (*p* < 0.05). No significant differences were observed in other amino acids between the groups (*p* > 0.05). The STH sheep have a more abundant amino acid content, which leads to different tastes of meat, compared to the crossbred sheep. The details are shown in Supplementary Table 4.

**Table 5:** The amino acid composition between the groups.

**Amino acid categories**	**Amino acids (g/100g)**	**Breeds (Mean** ±**SEM)**		
		**STH sheep (*****n** =* **6)**	**Do** × **STH sheep (*****n** =* **6)**	**Au** × **STH sheep (*****n** =* **6)**
Non-essential	Asp	7.77 ± 0.05^a^	7.44 ± 0.10^b^	7.44 ± 0.10^b^
	Arg	4.71 ± 0.40	5.12 ± 0.10	5.25 ± 0.10
	His	3.46 ± 0.09	3.34 ± 0.09	3.54 ± 0.09
	Ser	3.45 ± 0.03	3.30 ± 0.06	3.27 ± 0.06
	Glu	15.25 ± 0.26	14.84 ± 0.23	14.59 ± 0.19
	Pro	2.17 ± 0.11	2.18 ± 0.05	2.08 ± 0.05
	Gly	3.67 ± 0.09	3.65 ± 0.04	3.50 ± 0.05
	Ala	4.99 ± 0.11^a^	4.51 ± 0.05^b^	4.46 ± 0.06^b^
	Tyr	3.24 ± 0.05	3.11 ± 0.05	3.07 ± 0.06
Essential	Val	4.11 ± 0.03	3.87 ± 0.07	3.90 ± 0.08
	Met	1.83 ± 0.04	1.81 ± 0.03	1.89 ± 0.04
	Ile	3.83 ± 0.01^a^	3.57 ± 0.06^b^	3.61 ± 0.05^b^
	Leu	6.98 ± 0.04^a^	6.65 ± 0.11^b^	6.60 ± 0.08^b^
	Phe	3.27 ± 0.11	3.36 ± 0.05	3.50 ± 0.09
	Lys	7.58 ± 0.09^a^	7.05 ± 0.12^b^	7.10 ± 0.09^b^
	Thr	3.93 ± 0.04^a^	3.79 ± 0.06^ab^	3.72 ± 0.06^b^
Non-essential amino acid		48.71 ± 0.81	47.50 ± 0.71	47.21 ± 0.66
Essential amino acid		31.51 ± 0.15^a^	30.10 ± 0.48^b^	30.34 ± 0.44^ab^

STH, Small-tailed Han sheep; Do × STH, Dorper and small-tailed Han crossbred; Au × STH, Australian white and small-tailed Han crossbred. ^a, b^different superscript letters indicate significant differences among groups (*P* < 0.05).

### Volatile compound profiles

The content and composition of volatile compounds influence the flavor of mutton (37). To verify whether the crossbred sheep had different mutton odors compared to the STH sheep, a sample was taken from the semitendinosus muscle to compare the content and composition of the volatile chemical substances among the groups. A total of 46 volatile compounds were detected in the test, but only 29 volatile compounds had complete data in the groups. The results showed that there were no significant differences in the volatile compounds between the groups (*p* > 0.05) (Table 6). However, decanal was only detected in the Au × STH sheep, methyl decanoate was only detected in the STH sheep, methyl arachidonate was only detected in the Do × STH sheep, and ethyl palmitoleate was only detected in the crossbred sheep. An odor activity value (OAV) was calculated using the odor threshold (38). The results showed that heptanal, octanal, nonanal, decanal, and dodecanal volatile compounds may provide more abundant flavors in the Au × STH sheep due to a high OAV value. The OAV value of the tetradecanal compound in the STH and Do × STH sheep was more than 1, whereas in the Au × STH sheep, the value was < 1. The high OAV value of 1-octen-3-ol may affect the flavor of mutton of the STH sheep. The volatile compound composition and the OAV value may lead to different flavors of mutton between the groups. The details are shown in Supplementary Table 5.

**Table 6:** The volatile compounds composition between the groups.

**Categories**	**Volatile compounds (μg/10 g)**	**Threshold (μg/kg)**	**Breeds (Mean** ±**SEM)**					
			**STH sheep (*****n** =* **6)**	**OAV**	**Do** × **STH sheep (*****n** =* **6)**	**OAV**	**Au** × **STH sheep (*****n** =* **6)**	**OAV**
Aldehydes	Hexanal	4.5	0.69 ± 0.24	15.44 ± 5.42	0.55 ± 0.18	12.18 ± 3.92	1.10 ± 0.42	18.83 ± 9.77
	Heptanal	3	0.24 ± 0.06	8.11 ± 1.97	0.17 ± 0.08	5.53 ± 2.70	0.70 ± 0.35	23.48 ± 11.74
	Benzaldehyde	350	0.40 ± 0.07	0.11 ± 0.02	0.15 ± 0.05	0.04 ± 0.01	0.48 ± 0.16	0.14 ± 0.04
	Octanal	0.7	0.33 ± 0.02	47.51 ± 2.29	0.38 ± 0.12	54.11 ± 16.94	0.85 ± 0.31	121.85 ± 43.67
	Nonanal	1	0.61 ± 0.13	61.40 ± 13.18	0.37 ± 0.10	36.79 ± 9.52	1.05 ± 0.50	104.73 ± 50.03
	Decanal	0.1	-	-	-	-	0.09 ± 0.02	91.2 ± 16.17
	Dodecanal	2	0.42 ± 0.28	20.93 ± 14.01	0.89 ± 0.73	44.74 ± 36.33	2.94 ± 1.22	147.14 ± 61.03
	Tetradecanal	64	0.70 ± 0.35	1.09 ± 0.54	1.17 ± 0.45	1.82 ± 0.70	0.36 ± 0.15	0.56 ± 0.23
Alcohols	Silanediol, dimethyl	-	4.34 ± 1.21		1.11 ± 0.34		2.99 ± 0.66	
	1-octen-3-ol	1.5	0.82 ± 0.28	54.51 ± 18.74	0.44 ± 0.24	29.43 ± 16.06	0.53 ± 0.13	35.07 ± 8.84
Esters	Methyl decanoate	4.3	0.24 ± 0.05	5.59 ± 1.11	-	-	-	-
	Ethyl decanoate	8.6	0.22 ± 0.08	2.53 ± 0.92	0.39 ± 0.13	4.51 ± 1.48	0.29 ± 0.07	3.37 ± 0.79
	Methyl dodecanoate	-	1.18 ± 0.11		0.58 ± 0.19		1.69 ± 0.54	
	Methyl myristate	-	1.17 ± 0.26		1.31 ± 0.39		2.90 ± 0.76	
	Ethyl myristate	4,000	0.20 ± 0.09	0.005 ± 0.002	0.28 ± 0.11	0.007 ± 0.003	0.37 ± 0.12	0.009 ± 0.003
	Methyl Pentadecanoate	-	0.82 ± 0.44		0.74 ± 0.38		0.74 ± 0.26	
	Methyl palmitate	2,000	1.57 ± 1.13	0.08 ± 0.06	2.17 ± 1.50	0.11 ± 0.07	2.25 ± 1.33	0.11 ± 0.07
	Ethyl palmitate	2,000	0.63 ± 0.33	0.03 ± 0.02	1.63 ± 0.69	0.08 ± 0.03	0.60 ± 0.29	0.03 ± 0.01
	Methyl palmitoleate	-	0.41 ± 0.05		0.45 ± 0.19		0.58 ± 0.15	
	Ethyl palmitoleate	-	-		0.19 ± 0.04		0.26 ± 0.07	
	Methyl margarate	-	0.21 ± 0.03		0.30 ± 0.14		0.14 ± 0.04	
	Methyl stearate	-	1.68 ± 0.43		2.03 ± 0.54		1.09 ± 0.24	
	Ethyl stearate	-	0.25 ± 0.08		0.64 ± 0.23		0.21 ± 0.09	
	Methyl oleate	-	4.34 ± 0.50		5.10 ± 1.33		6.83 ± 2.47	
	Ethyl oleate	-	0.62 ± 0.20		2.18 ± 0.67		1.20 ± 0.44	
	Methyl linoleate	-	1.58 ± 0.42		2.05 ± 0.65		1.17 ± 0.24	
	Ethyl linoleate	-	0.50 ± 0.37		0.95 ± 0.53		0.17 ± 0.06	
	Methyl arachidonate	-	-		0.18 ± 0.10		-	
Monoterpenes	Limonene	100	0.50 ± 0.15	0.50 ± 0.15	0.30 ± 0.11	0.30 ± 0.11	0.24 ± 0.12	0.24 ± 0.12

STH, Small-tailed Han sheep; Do × STH, Dorper and small-tailed Han crossbred; Au × STH, Australian white and small-tailed Han crossbred.

### *The PDK4* and *TMEM273* gene expression are closely associated with IMF and fatty acid composition in the Do × STH crossbred sheep

**Figure 3:**
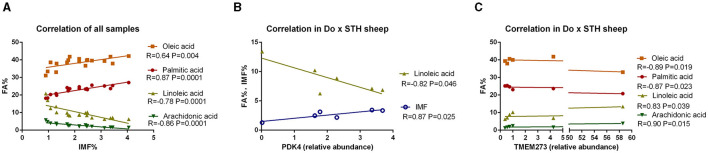
The correlation of the IMF content, genes expression, and fatty acid composition in the sheep. **(A)** The correlation of the IMF content and fatty acid composition; R represents the correlation coefficient. A *p*-value of <0.05 represents significant differences. **(B)** The correlation of the *PDK4* gene expression in the MLD with the IMF content and fatty acid composition in the Do × STH crossbred sheep. R represents the correlation coefficient. A *p*-value of <0.05 represents significant differences. **(C)** The correlation of the *TMEM273* gene expression in the MLD with the fatty acid composition in the Do × STH crossbred sheep. R represents the correlation coefficient. A *p*-value of <0.05 represents significant differences.

Many studies have reported that IMF content has a close relationship with fatty acid composition in livestock (39, 40); thus, the relationship was compared in this study. The results showed that the IMF content was positively associated with palmitic acid and oleic acid and was negatively correlated with linoleic acid and arachidonic acid in all samples (*p* < 0.05) (Figure 3A). The results were as expected. Many studies have demonstrated that the *PPARG* gene regulates adipogenesis and lipid metabolism (41). Our previous experiments found that the *PDK4* gene affects fat accumulation (42). The *PPARG* and *PDK4* gene expression in the MLD was detected by conducting the qPCR test. The qPCR test results showed no significant differences in the *PDK4* and *PPARG* gene expression between the groups (Table 7) (*p* > 0.05). The correlation coefficient of the gene expression and IMF content or fatty acid composition were compared. The results showed that the *PPARG* and *PDK4* gene expression had no association with the IMF content and fatty acid content in the STH sheep (*p* > 0.05); in the Au × STH offspring, the correlation trends were similar to those in the STH sheep (*p* > 0.05). Furthermore, the *PDK4* gene expression was positively correlated with the IMF content and was negatively correlated with the linoleic acid content in the Do × STH offspring (*p* < 0.05) (Figure 3B). Our previous experiments found that the *TMEM273* gene was negatively regulated by the *PDK4* gene and affected lipid metabolism. The correlation of the *TMEM273* gene expression in the MLD with the IMF content or fatty acid composition was compared, and the results showed that the *TMEM273* gene expression had no association with the IMF content (R = −0.783, *P* = 0.066) in the Do × STH offspring (Table 8). However, the *TMEM273* gene expression had a positive association with the linoleic acid and arachidonic acid contents and a negative correlation with the oleic acid and palmitic acid contents in the Do × STH offspring (*p* < 0.05) (Figure 3C). The details are shown in Supplementary Tables 6–9.

**Table 7:** qPCR results between the groups.

**Genes**	**Breeds (Mean** ±**SEM)**		
	**STH sheep (*****n** =* **6)**	**Do** × **STH sheep (*****n** =* **6)**	**Au** × **STH sheep (*****n** =* **6)**
PPARG	8.78 ± 6.11	19.17 ± 6.37	8.08 ± 2.91
PDK4	1.31 ± 0.60	2.12 ± 0.54	1.13 ± 0.58

STH, Small-tailed Han sheep; Do × STH, Dorper and small-tailed Han crossbred; Au × STH, Australian white and small-tailed Han crossbred.

**Table 8:** The correlation of the IMF content with the gene expression and fatty acid composition.

**Sheep**	**IMF**	**Gene expression**		**Fatty acids**				
		**PPARG**	**PDK4**	**Palmitic acid**	**Palmitoleic acid**	**Oleic acid**	**Linoleic acid**	**Arachidonic acid**
STH sheep (*n =* 6)	Correlation	0.548	−0.452	0.958^**^	0.954^**^	0.864^*^	−0.875^*^	−0.919^**^
	*p-*value	0.261	0.368	0.003	0.003	0.026	0.022	0.01
Au × STH sheep (*n =* 6)	Correlation	−0.424	0.422	0.828^*^	0.554	0.764	−0.851^*^	−0.816^*^
	*p*-value	0.402	0.404	0.042	0.254	0.077	0.032	0.048

STH, Small-tailed Han sheep; Do × STH, Dorper and small-tailed Han crossbred; Au × STH, Australian white and small-tailed Han crossbred. ^*^P < 0.05; ^**^P < 0.01.

## Discussion

Small-tailed Han sheep, an indigenous breed in China, are known for their high reproductive ability and resistance to crude feed; in the past, their characteristics met the demand of local markets, but they are no longer suitable for the current market condition (43). Recently, the objectives of breeding sheep have become more focused on improving meat yield and quality. Crossbreeding improves the offspring's production performance. Dorper and Australian White rams are generally crossbred with STH ewes to improve the offspring's meat yield and quality in Northeast China. However, the advantages and disadvantages of crossbred sheep in terms of slaughter and meat quality traits remain unclear. The experiment in this study aimed to provide detailed data to reveal the advantages and disadvantages of crossbred sheep, identify the candidate genes related to meat quality traits, and find ways to improve the traits.

In this study, the slaughter traits of the crossbred sheep showed higher levels than the STH sheep, as expected. The Dorper × Mongolian crossbred sheep also showed higher carcass weight and a faster growth rate than the Mongolian sheep under the same feeding condition (44). We observed a phenomenon where the STH sheep were more active, while the crossbred sheep preferred to remain stationary during the feeding period. In addition, the partial nutrition uptake maintained the tail growth in the STH sheep (Table 2). It may be one of the factors that lead to the STH sheep having lower weight and a slower growth rate. A study reported that the body weight of 6-month-old Dorper sheep reached over 60 kg (45). Even though the experimental sheep were fed under different feeding conditions, the results still demonstrated that the slaughter traits of the crossbred sheep were lower than those of the pure Dorper sheep. Red Maasai × Dorper crossbred sheep also showed similar trends (46). The slaughter traits of the crossbred sheep were higher than those of the STH sheep but were lower than those of pure sheep breeds; therefore, improving the traits became an important breeding objective.

The intramuscular fat, protein, and moisture are the main components of the muscular *longissimus dorsi* (47), which not only play crucial roles in body activities and development but also affect the mutton flavor, tenderness, juiciness, and nutrition (48). In this study, the crossbred sheep had higher IMF content but different protein and moisture contents. Over 20 g of protein per 100 g of meat belongs to lean meat (49). The results demonstrated that the Au × STH sheep possessed leaner mutton. The moisture content affects mutton color and tenderness (49). We found that the Do × STH sheep had brighter and redder meat, as expected. The pH value influenced by the muscle glycogen glycolysis affects the meat color and cooking percentage (50, 51). The higher pH value and moisture content may have led to the highest cooking percentage in the Do × STH sheep. In contrast, the Do × STH sheep may have had better meat quality than the Au × STH sheep.

Fatty acids are essential components of triglycerides stored within the adipocyte (52); they play vital physiological functions and affect animal health (53). The content of palmitic acid, stearic acid, and oleic acid in this study summed up to be approximately 80%, which was consistent with the ruminants' fatty acid composition (54). Given that saturated fatty acids improve adipocyte differentiation and fat accumulation (55), higher contents of myristate acid, palmitic acid, and margaric acid and higher IMF were all observed in the crossbred sheep. The results are consistent with the above reports. Arachidonic acid inhibits adipocyte differentiation and fat accumulation (56); the results of this study also showed that the STH sheep had the highest content of arachidonic acid and the lowest IMF content. The content of saturated and monounsaturated fatty acids is positively related to meat flavor (57); the results of this study indicated that the Do × STH sheep may have better meat flavor. However, excessive saturated fatty acid uptake may lead to a high risk of obesity and related diseases in humans (58–61). Fatty acids have different heritability (62). A possible efficient way to alleviate the negative effects is to reduce the saturated fatty acid content and increase the monounsaturated fatty acid content through molecular breeding in the Do × STH sheep.

Amino acid content and composition affect meat nutrition and quality (63), and diverse amino acids possess different tastes during cooking. For example, Glu, Arg, Asp, and Gly amino acids provide a umami taste (64), Gly, Ser, Thr, Lys, Pro, and Ala amino acids provide a sweet taste (65), and Leu, Ala, and Arg amino acids provide aromatic flavor to the meat (66). A study reported that Dorper sheep have lower amino acid content, a finding that aligns with the results observed in our study for the Do × STH sheep (44). However, the higher content of the amino acids in the STH sheep may contribute to a richer taste compared to the crossbred sheep.

Fatty acids and amino acids form different kinds of volatile compounds during cooking, which brings more abundant and complex sensory feelings (67). Aldehydes, a species of intermediates from the Maillard reaction and lipid oxidation (68), are the main components of volatile substances (69). Aldehydes possess a fatty aroma, but their higher concentrations lead to a rancid odor (70, 71). Heptanal has fatty, citrusy, and rancid odors; octanal has soapy, lemony, and green odors; nonanal has citrusy and green odors; and decanal has a herbal scent (72). The value of the OAV demonstrated that the Au × STH sheep may have more abundant odors. Few alcohols were identified in this study, among which 1-Octen-3-ol had fresh and mushroom-like odors (73). Additionally, fewer short-chain fatty acid esters were detected, which could be attributed to their low volatility and associated with fruity flavors (74). Moreover, methyl decanoate released a coconut odor in the STH sheep. Most long-chain fatty acid esters with oily, flowery, and tee odors were identified, but they had unclear or higher odor thresholds (75, 76). Limonene, a lemon odor, was found in all sheep breeds (77). The results demonstrated that the mutton in the three breeds may have different meat odors.

Palmitic and oleic acids promote fat accumulation, but linoleic and arachidonic acids inhibit triglyceride deposition. In this study, the correlation of the IMF content and fatty acid content was consistent with the previous reports (62, 78–81), which indicates that the results are reliable. The *PDK4* gene positively affects adipogenesis and fat accumulation. Hence, the correlations of the *PDK4* gene expression with the IMF content and fatty acid content were found to be consistent with the existing reports (42). The genetic background of the Do × STH sheep is different from that of other sheep; thus, similar results could not be observed in the STH sheep or Au × STH sheep. Our previous experiments found that the *PDK4* gene negatively regulated the *TMEM273* gene, making the correlations of the *TMEM273* gene expression with the IMF content and fatty acid content logical. With an increasing number of validated experimental animals, the expression of the *PDK4* and *TMEM273* genes may act as an indicator for evaluating the IMF content in Do × STH sheep.

## Conclusion

The Do × STH offspring had higher meat yield and quality. The three sheep had different mutton flavors. The *PDK4* and *TEME273* genes had close correlations with the fatty acid composition and IMF content, and they could be considered as potential candidates for detecting or improving the IMF content in Do × STH crossbred sheep.

## Data availability statement

The original contributions presented in the study are included in the article/Supplementary material, further inquiries can be directed to the corresponding author.

## Ethics statement

All procedures involving animals such as welfare and ethical issues were approved by the Ethics Committee of Jilin Academy of Agricultural Sciences (AWEC 2019A05, 16 May 2019). The studies were conducted in accordance with the local legislation and institutional requirements. Written informed consent was obtained from the owners for the participation of their animals in this study.

## Author contributions

CX: Data curation, Formal analysis, Investigation, Methodology, Project administration, Software, Validation, Visualization, Writing – original draft, Writing – review & editing. YLiu: Data curation, Methodology, Writing – review & editing. WZ: Data curation, Methodology, Validation, Writing – review & editing. YLia: Data curation, Methodology, Validation, Writing – review & editing. CC: Data curation, Methodology, Writing – review & editing. SY: Data curation, Methodology, Writing – review & editing. WF: Data curation, Formal analysis, Software, Writing – review & editing. LM: Data curation, Investigation, Methodology, Writing – review & editing. ZY: Data curation, Investigation, Methodology, Resources, Writing – review & editing. ZL: Data curation, Investigation, Methodology, Writing – review & editing. BZ: Investigation, Resources, Software, Visualization, Writing – review & editing. ZZ: Data curation, Methodology, Resources, Writing – review & editing. LZ: Investigation, Project administration, Software, Writing – review & editing. HM: Data curation, Methodology, Resources, Writing – review & editing. HJ: Funding acquisition, Project administration, Resources, Supervision, Writing – review & editing. YC: Funding acquisition, Investigation, Project administration, Resources, Supervision, Writing – review & editing.
